# Editorial for the Special Issue on State-of-the-Art CMOS and MEMS Devices

**DOI:** 10.3390/mi15030327

**Published:** 2024-02-27

**Authors:** Zhiming Chen

**Affiliations:** School of Integrated Circuits and Electronics, Beijing Institute of Technology, Beijing 100081, China; czm@bit.edu.cn

## 1. Introduction

Complementary Metal Oxide Semiconductor (CMOS) and Micro-Electro-Mechanical System (MEMS) devices play significant roles in emerging research fields such as artificial intelligence (AI) [1], 5G communication [2,3], the Internet of Things (IoT) [4], and satellite communication [5,6]. With the continuous development of material science and micro-fabrication processes, high-performance CMOS and MEMS devices have been the subject of extensive investigations and have advanced significantly [7,8]. In recent years, the rapid development of heterogeneous integration [9] and complex multi-functional systems has resulted in higher requirements for CMOS and MEMS devices; it is thus urgent to develop novel CMOS integrated circuits (ICs) and MEMS devices utilizing advanced materials, techniques, and processes. Thus, the research in this field is currently focused on the state-of-the-art design [10,11,12], modeling [13], fabrication [14], and application of CMOS and MEMS devices [15,16]. 

This Special Issue, entitled “State-of-the-Art CMOS and MEMS Devices”, presents crucial research progress in the area of advanced CMOS ICs and MEMS devices. The Special Issue contains 10 published articles (9 research articles and 1 review article) that address the urgent demand for high-performance silicon ICs and MEMS devices; these articles aim to further advance the field through extensive explorations of device and circuit design techniques, performance optimization methods, and system integration strategies.

## 2. Overview of the Published Articles

The development of software-defined radio, 5G mobile communication, satellite communication, and wireless sensor networks has augmented the demands for high-performance CMOS radio frequency (RF) transceiver front-ends and their key building blocks, such as low-noise amplifiers (LNAs), power amplifiers (PAs), and circulators. To enable the utilization of multi-standard wireless communication systems that support several applications simultaneously, Wan et al. (contribution 1) proposed the application of an inductorless and gain-controllable 0.5~2.5 GHz wideband LNA based on second-generation current-controlled current conveyors (CCCIIs). By varying the DC biasing current of the CCCII, the voltage gain of the proposed LNA is controllable in the range of 1~18 dB. The design achieves a typical voltage gain of 12.6 dB with a gain ripple of ±1.5 dB, a noise figure of 4.05~4.35 dB across the whole band of 0.5~2.5 GHz, and an input third-order intercept point (IIP3) of −2.5 dBm at 1.5 GHz. By utilizing a 0.18 μm RF CMOS, the LNA occupies an active chip area of 0.096 mm^2^, with a power consumption of 12.0 mW.

Because the specification of PAs is closely related to variations in the humidity, Zhou et al. (contribution 2) conducted an experimental study of PA specifications under different humidity conditions to illustrate the relationship between the degradation of PA performance and humidity conditions. The paper presents results regarding the degradation of a PA subjected to different humidity levels, where the S_21_ and output power decrease with the increase in humidity. Furthermore, it also analyzes the main reason for this degradation, which is the decrease in the oxide capacitance and increase in the threshold voltage as the humidity increases, resulting in a reduction in the transconductance and an increase in the on-resistance.

For those who are seeking a broad overview and a general understanding of the design of PAs in CMOS technology, the review article by Hamid et al. (contribution 3) could be a good reference. The authors provide a state-of-the-art review on CMOS RF PAs for wireless communication systems. RF PAs in a CMOS transmitter play a crucial role in amplifying RF signals and transmitting them through the antenna. This review paper presents a concise discussion of the performance metrics, which is followed by an overview of the research trends observed in CMOS PA design techniques with a focus on efficiency, linearity, and bandwidth enhancement. Although the on-chip transformer and out-phasing techniques are capable of delivering the watt-level maximum output power for CMOS PA, they are still limited in delivering the watt-level linear output power.

Full-duplex (FD) communication enables simultaneous transmission and reception; however, the implementation of on-chip non-magnetic passive circulators is a great challenge. Gao et al. (contribution 4) designed and implemented a non-magnetic on-chip passive circulator operating at the Ku band in a 90 nm bulk CMOS using switch-based spatio-temporal conductivity modulation. Owing to the four-phase non-overlapping clock signal, the proposed circulator achieves a 3.9 dB transmitter (TX)-to-antenna (ANT) and a 4.0 dB ANT-to-receiver (RX) insertion loss. The TX-to-RX isolation is better than 17.2 dB, and the TX-to-ANT IIP3 and ANT-to-RX IIP3 values are 19.7 dBm and 20.0 dBm, respectively, while occupying a die area of 1.55 mm × 1.15 mm. To further develop CMOS ICs in a millimeter-wave range, He et al. (contribution 5) present a D-band direct conversion IQ receiver with an on-chip multiplier chain. The D-band LNA is implemented using a gain-boosting and stagger-tuning technique to provide high gain and a large bandwidth. The X9 multiplier chain including Marchand balun and a quadrature (90°) hybrid is employed to provide four LO signals to drive the IQ mixer. This receiver is implemented in a 130 nm SiGe process and consumes a core area of 1.04 mm^2^. The proposed receiver exhibits a 20 GHz bandwidth from 150 GHz to 170 GHz, with a conversion gain of 28 dB and NF of 7.3 dB at 158 GHz.

Yang et al. (contribution 6) present an ultra-low-power analog multiplier–divider that is compatible with digital code words and is applicable to the integrated structure of resistive random-access memory (RRAM)-based computing-in-memory (CIM) macros. The multiplication and division of the current are accomplished by a current-mirror-based structure, improving the energy efficiency. Designed and fabricated in a 55 nm CMOS process, the proposed work achieves 8-bit precision for analog current multiplication and division operations. The measurement results show that the signal delay is 1 μs when performing the 8-bit operation. The power consumption is less than 6.15 μW with a 1.2 V supply voltage.

The increasing complexity of the power supply network has led to the susceptibility of the power supply port to electromagnetic interference (EMI). In order to obtain a deep understanding of the electromagnetic susceptibility (EMS) mechanism and guide the design of the electromagnetic compatibility (EMC) of operational amplifiers (op-amps), Huang et al. (contribution 7) assessed the effect of EMI on the performance of op-amps through the power supply port by employing a bulk current injection (BCI) method. The authors proposed a novel method of conducted susceptibility and obtained the susceptibility threshold regularities of the op-amps at the power supply port under the interference, which provided evidence that EMI reduced the reliability of the op-amp by affecting the offset voltage of op-amps; this also demonstrated that the sensitivity type of op-amps is peak sensitive at the power supply port.

Hot-carrier degradation (HCD) has been recognized as the most harmful degradation issue limiting the lifetime of modern metal oxide semiconductor field-effect transistors (MOSFETs). Tyaginov et al. (contribution 8) developed a compact physical model for HCD that is valid over a wide range of gate–source (V_gs_) and drain–source voltages (V_ds_) based on the refined modeling of the carrier transport for both primary and secondary carriers. Special attention is paid to the contribution of secondary carriers (generated by impact ionization) to HCD, which is shown to be significant under stress conditions with low V_gs_ and relatively high V_ds_. To validate the model, foundry quality n-channel transistors were employed, with a broad range of stress voltages.

To facilitate the integration of RF ICs and MEMS devices and minimize the form factor of electronic systems, Dong et al. (contribution 9) propose novel integrated passive devices (IPDs) that include capacitors, inductors, and a bandpass filter designed by employing through-silicon via (TSV) structures. The influence of the structural parameters of TSVs on the electrical performance of the TSV-based capacitor and inductor are evaluated. Moreover, a compact third-order Butterworth bandpass filter with a central frequency of 2.4 GHz is developed, and the footprint is only 0.814 mm × 0.444 mm. The simulated 3 dB bandwidth of the filter is 410 MHz, and the fraction bandwidth (FBW) is 17%. In addition, the in-band insertion loss is less than 2.63 dB, and the return loss in the passband is better than 11.4 dB, thus exhibiting good RF performance.

With the development of space laser communication and satellite internet constellations, the demand for microminiature laser communication terminals is expanding. To meet the requirements of size, weight and power (SWaP), miniaturized terminals require smaller drive components in order to complete on-orbit scanning and capture. Wang et al. (contribution 10) propose a laser scanning capture model based on a MEMS micromirror that can provide rapid, large-scale scanning analysis. A scanning overlap factor is introduced to enhance the likelihood of capture under the influence of microvibrations. An experimental analysis validated the effectiveness of the scanning capture strategy, thus establishing a theoretical basis for future ultra-long-distance microspace laser communication.

## 3. Conclusions

The articles published in this Special Issue present important advancements in the field of CMOS and MEMS devices. I would like to express my sincere gratitude to all the authors, who provided insights and shared their solutions, and I also would like to thank the editors and reviewers who helped to improve the papers and made important contributions to this Special Issue. However, several challenges and obstacles remain to be addressed in the future, such as ultra-wideband CMOS transceivers, CMOS-MEMS integration technology, and high-density multi-chiplet 2.5D/3D-integrated microsystems, etc. We hope that the articles showcased in this Special Issue are interesting and inspiring for its readers, especially young scholars who are eager to learn about recent advances and contribute future research in the field.

## List of Contributions

1. Wan, Q.; Liu, J.; Chen, S. An Inductorless Gain-Controllable Wideband LNA Based on CCCIIs. *Micromachines* **2022**, *13*, 1832. https://doi.org/10.3390/mi13111832.
2. Zhou, S.; Yang, C.; Wang, J. Experimental Investigation of Relationship between Humidity Conditions and Degradation of Key Specifications of 0.1–1.2 GHz PA in 0.18 μm CMOS. *Micromachines* **2022**, *13*, 1162. https://doi.org/10.3390/mi13081162.
3. Hamid, S.S.; Mariappan, S.; Rajendran, J.; Rawat, A.S.; Rhaffor, N.A.; Kumar, N.; Nathan, A.; Yarman, B.S. A State-of-the-Art Review on CMOS Radio Frequency Power Amplifiers for Wireless Communication Systems. *Micromachines* **2023**, *14*, 1551. https://doi.org/10.3390/mi14081551.
4. Gao, J.; Wang, X.; Han, F.; Wan, J.; Gu, W. Analysis and Design of a Non-Magnetic Bulk CMOS Passive Circulator Using 25% Duty-Cycle Clock. *Micromachines* **2023**, *14*, 33. https://doi.org/10.3390/mi14010033.
5. He, F.; Ding, Y.; Xu, Z.; Ni, M.; Tian, Y.; Zhang, Z.; Shi, Z.; Wang, K.; Xie, Q.; Wang, Z. A D-Band Direct-Conversion IQ Receiver with 28 dB CG and 7.3 dB NF in 130 nm SiGe Process. *Micromachines* **2023**, *14*, 87. https://doi.org/10.3390/mi14010087.
6. Yang, Y.; Lv, S.; Li, X.; Wang, X.; Wang, Q.; Yuan, Y.; Liang, S.; Zhang, F. An Ultra-Low-Power Analog Multiplier–Divider Compatible with Digital Code for RRAM-Based Computing-in-Memory Macros. *Micromachines* **2023**, *14*, 1482. https://doi.org/10.3390/mi14071482.
7. Huang, P.; Li, B.; Wei, M.; Hao, X.; Chen, X.; Huang, X.; Huang, W.; Zhou, S.; Wen, X.; Xie, S.; et al. Electromagnetic Susceptibility Analysis of the Operational Amplifier to Conducted EMI Injected through the Power Supply Port. *Micromachines* **2024**, *15*, 121. https://doi.org/10.3390/mi15010121.
8. Tyaginov, S.; Bury, E.; Grill, A.; Yu, Z.; Makarov, A.; Keersgieter, A.D.; Vexler, M.; Vandemaele, M.; Wang, R.; Spessot, A.; et al. Compact Physics Hot-Carrier Degradation Model Valid over a Wide Bias Range. *Micromachines* **2023**, *14*, 2018. https://doi.org/10.3390/mi14112018.
9. Dong, H.; Ding, Y.; Wang, H.; Pan, X.; Zhou, M.; Zhang, Z. Design of a Novel Compact Bandpass Filter Based on Low-Cost through-Silicon-Via Technology. *Micromachines* **2023**, *14*, 1251. https://doi.org/10.3390/mi14061251.
10. Wang, X.; Han, J.; Wang, C.; Xie, M.; Liu, P.; Cao, Y.; Jing, F.; Wang, F.; Su, Y.; Meng, X. Beam Scanning and Capture of Micro Laser Communication Terminal Based on MEMS Micromirrors. *Micromachines* **2023**, *14*, 1317. https://doi.org/10.3390/mi14071317.
